# Multisystem inflammatory syndrome in children and Kawasaki disease; comparison of their clinical findings and one-year follow-up—a cross-sectional study

**DOI:** 10.1186/s13052-023-01489-6

**Published:** 2023-07-21

**Authors:** Saghar Mehrban, Fatemeh Tahghighi, Ehsan Aghaei Moghadam, Vahid Ziaee

**Affiliations:** 1 Children’s Medical Center, Pediatrics Center of Excellence, Tehran University of Medical Sciences, Tehran, Iran; 2 Department of Pediatrics, Tehran University of Medical Sciences, Tehran, Iran; 3 Pediatric Rheumatology Research Group, Rheumatology Research Center, Tehran University of Medical Sciences, Tehran, Iran

**Keywords:** Coronary aneurysm, COVID-19, Kawasaki disease, Mucocutaneous Lymph Node Syndrome, Pediatric multisystem inflammatory disease

## Abstract

**Background:**

Studies on Multisystem Inflammatory Syndrome in Children (MIS-C) and Kawasaki Disease (KD) have yielded inconsistent results and are lacking in Asian and African countries. This study aimed to compare the laboratory and clinical features, short-term outcomes, and one-year follow-ups of a large cohort of MIS-C and KD patients.

**Methods:**

Data from 176 MIS-C and 56 KD patients admitted to Tehran Children's Medical Center between January 2021 and January 2022 were collected. Patients were followed up until January 2023.

**Results:**

While lymphopenia and thrombocytopenia were more prevalent in MIS-C (73.2% vs. 20% in KD, *p* < 0.001), KD patients exhibited a higher median white blood cell count and prevalence of anemia, along with higher fibrinogen and erythrocyte sedimentation rate levels (*p* < 0.001, p < 0.001, *p* = 0.005, *p* < 0.001, respectively). MIS-C patients also exhibited lower ejection fraction, a greater occurrence of pericardial effusion, and a higher incidence of coronary aneurysms and ectasia, and ascites. Echocardiography after seven days of treatment showed a reduction in pathologies for both groups, but it was significant only for MIS-C. After one year, coronary artery abnormalities remained in only six cases.

**Conclusions:**

In conclusion, this study highlights differences between MIS-C and KD, including laboratory indices as well as echocardiographic and abdominal ultrasound findings. These findings contribute valuable data on Iranian patients to the existing literature on this topic and have significant implications for accurate diagnosis and improved management of pediatric patients presenting with these conditions.

## Background

The globally known Coronavirus Disease 2019 (COVID-19) primarily affects the respiratory tract, resulting in severe pneumonia. Although it can affect individuals of all ages, the disease was initially assumed to present with mild symptoms among most children [1]. However, in May 2020, several reports of an increase in Kawasaki-like manifestations among children with COVID-19 raised attention to a novel febrile hyper-inflammatory syndrome associated with SARS-CoV-2 infection resembling the features of Kawasaki Disease (KD) [2, 3]. The disease was later entitled Multisystem Inflammatory Syndrome in Children (MIS-C). Both the World Health Organization (WHO) and the Centers for Disease Control and Prevention (CDC) proposed case definition criteria for its diagnosis, consisting of an age interval, involvement of body organs, evidence of COVID-19 exposure, and exclusion of other potential diagnoses [4, 5].

Many reports from different countries have been published to reveal the differences between KD and MIS-C. For instance, KD is more prevalent in children below five, whereas MIS-C is usually seen in older children [6]. Regarding cardiac manifestations, pericardial and myocardial involvements are commonplace in MIS-C, while coronary aneurysms and ectasia are more frequently detected in KD patients. Lymphopenia and elevated inflammatory markers such as CRP, D-dimer, and ferritin are reported to be more common among patients with MIS-C [7].

Despite the severe outbreak of COVID-19 and the massive number of affected children since 2019 in the Middle East, especially in Iran, no comprehensive studies have been conducted to compare clinical findings among MIS-C and KD patients. Our study aimed to characterize these two entities using their laboratory, cardiac, and abdominal ultrasound findings, short-term outcomes after treatment, and one-year follow-ups.

## Methods

### Case definition

This cross-sectional study was conducted at the Tehran Children's Medical Center in Tehran, Iran, between January 1^st^, 2021, and January 1^st^, 2022. Patients who were hospitalized with a first impression of MIS-C or KD were included in this study. Selected patients were followed up until January 1^st^, 2023.

The diagnostic criteria of the CDC were used to define MIS-C cases. According to the CDC's criteria, children under 21 years of age who presented with fever, evidence of inflammation in their laboratory markers, severe illness requiring hospitalization, and involvement of two or more body organs (cardiac, renal, respiratory, hematologic, gastrointestinal, dermatologic, or neurological involvements) were diagnosed with MIS-C. Patients also had no probable alternative diagnosis and met the criteria for SARS-CoV-2 exposure (e.g., close-contact exposure to a known patient with COVID-19, evidence of SARS-CoV-2 infection by PCR, serology, or antigen test within four weeks prior to the initiation of symptoms) [5].

According to the AHA's criteria for KD diagnosis, any child with at least five days of fever, presenting four or more of the following clinical signs (rash, cervical lymphadenopathy of at least 1.5 cm in diameter, conjunctivitis, mucosal change in the oral cavity, and extremity changes) was diagnosed with KD [8].

### Patient groups & ethics

**Fig. 1:**
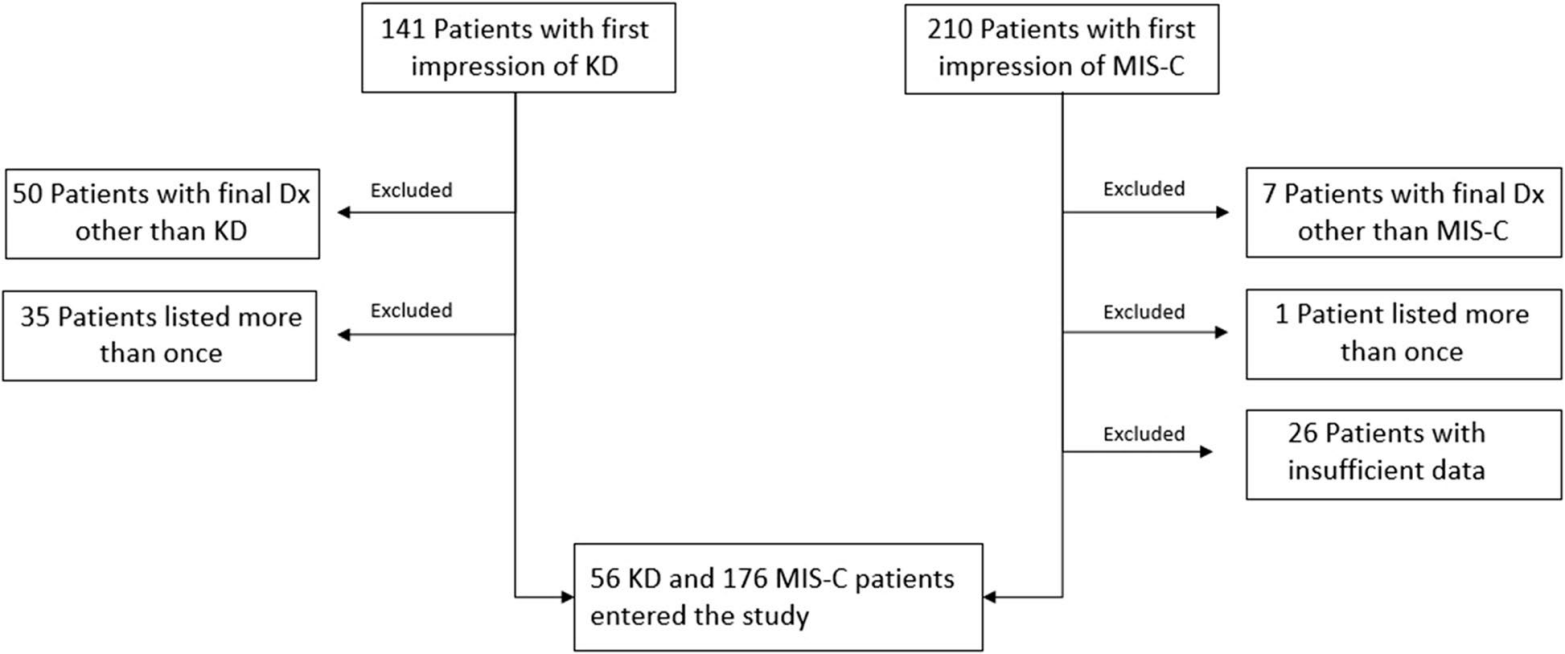
Summary of the study population. KD: Kawasaki disease, MIS-C: Multisystem inflammatory syndrome in children, Dx: Diagnosis

Overall, 141 and 210 patients were admitted with KD and MIS-C first diagnoses, respectively. Patients:

- 1) Who were hospitalized and listed more than once, with their second admission being less than two weeks apart from the first
- 2) whose final diagnosis was not similar to their first impression
- 3) with insufficient data on laboratory indices or echocardiography

were excluded from the study. The detailed number of excluded cases is depicted in Fig. 1.

All patients and their parents signed an informed consent at admission, indicating their agreement to use their clinical data anonymously for research purposes. The ethics committee approved this study and its methods at the Tehran University of Medical Sciences, Tehran, Iran (IR.TUMS.CHMC.REC.1399.134). All data were collected retrospectively from the medical records. Patients reported no past medical illnesses or previous hospitalizations. None of the patients were receiving medications before their admission.

### Clinical & laboratory evaluation

Complete blood count (CBC), including white blood cell count (WBC), lymphocyte count, platelet count (Plt), and hemoglobin concentration (Hb) levels, along with fibrinogen, ferritin, D-dimer, lactate dehydrogenase (LDH), creatine phosphokinase (CPK), creatine kinase myocardial band (CK-MB), troponin, erythrocyte sedimentation rate (ESR), and C-reactive protein (CRP) levels, were recorded using the patient's first blood test after admission. Reference values for laboratory indices were dependent on the kit used in the laboratory.

A single operator performed all echocardiograms using a GE Vivid E9 Ultrasound machine. Patients' first echocardiographic and abdominal US findings during hospitalization, the type of treatment each patient received, and their echocardiographic data 7 days after receiving their first treatment were also retrieved from medical records. Among echocardiographic findings, ejection fraction (EF) was reported in percentage. Decreased EF was defined as EF lower than 60%. The presence of pericardial effusion (PE), pericarditis, myocarditis, aortic valve insufficiency (AI), mitral valve insufficiency (MI), tricuspid valve insufficiency (TI), and pulmonary valve insufficiency (PI) was reported. Coronary artery abnormalities (CAAs) included ectasia and aneurysm. Ectasia refers to diffuse arterial dilatation in which the z-score of the arterial abnormality is between 2 and 2.5. An aneurysm is defined as a focal over 1.5-fold increase in arterial diameter, with a z-score of 2.5 or more [8, 9]. Follow-up echocardiography was performed for patients who exhibited any CAAs in any of their previous echocardiograms, 365 days after their first admission. Abdominal ultrasound findings were categorized into four groups: presenting with ascites, lymphadenopathy, both, or none.

AHA guidelines were used for KD treatment, according to which all KD patients were treated with IVIG (2 g/kg, single dose) and ASA (3–5 mg/kg once the fever subsided). IVIG-resistant patients were administered methylprednisolone pulse therapy (30 mg/kg/dose infused in 1–3 doses, followed by administration of oral prednisolone 2–3 weeks after discharge) [8]. MIS-C patients were treated based on American College of Rheumatology Clinical Guidance for Multisystem Inflammatory Syndrome in Children Associated With SARS-CoV-2 and Hyper inflammation in Pediatric COVID-19: Version 1 [10]. As per this protocol, severe MIS-C patients were treated with methylprednisolone pulse therapy (30 mg/kg/dose infused in 1–3 doses, followed by administration of oral prednisolone 2–3 weeks after discharge). If these patients had coronary artery involvement or myocarditis, IVIG (2 g/kg) was added to their therapeutic regimen. For patients requiring intensive care unit (ICU) care, enoxaparin was included in the treatment, while ASA (3–5 mg/kg/day) was added for other patients.

### Statistical analysis

Statistical analyses were performed using IBM SPSS Statistics version 26. Variables presenting normal distribution were compared using independent samples t-test and reported as mean-standard deviation (SD). For variables with kurtosis, we used the Mann–Whitney test, and the data were expressed using the median and first and third quartiles (Q1-Q3). Categorical variables were tested using cross-tabulation or logistic regression based on the number of comparisons. Paired samples t-test was utilized to compare EF values before and after treatment. The McNemar test was used to compare dichotomous variables before and after receiving treatment. *P*-values lower than 0.05 were considered statistically significant.

## Results

### Patients' demographics

This study included 176 patients in the MIS-C group and 56 patients in the KD group. The median age of patients was 41.5 months in the MIS-C group and 15.5 months in the KD group (*p* < 0.001). Additionally, 33.5% of MIS-C patients were above five years old, while this percentage was only 7.1% in the Kawasaki group (*p* < 0.001). There were no significant differences in gender composition between the two groups (*p* = 0.1). During hospitalization, 2 MIS-C patients expired due to disease severity, while none of the KD cases passed away (*p* = 0.4).

### Comparison of laboratory findings between studied groups

Thirteen laboratory indices were compared within two groups of patients*.* Table 1 depicts the significantly different laboratory findings among the studied groups. D-dimer and CRP levels were elevated in both groups, yet they did not exhibit any significant statistical difference.

**Table 1:** Comparison of patient groups regarding their laboratory findings

	**MIS-C**	**KD**	***P*-value**
**Laboratory findings**			
** WBC count (cells/µ**l)^**a**^	8220 (5300–12,192.5)	14,050 (9560–21,230)	** < 0.001**
** Lymphocyte count (cells/µ**l)^**a**^	1986.1 (1134.9–3149.3)	4800.6 (3209.3–6909)	** < 0.001**
** Lymphopenia n/N (%)**	123/168 (73.2%)	11/55 (20%)	** < 0.001**
Platelet count (cells^*^1000/µl)^**a**^	219.5 (151–303.7)	443 (337–573)	** < 0.001**
Thrombocytopenia **n/N (%)**	40/168 (23.8%)	3/55 (5.5%)	**0.003**
Hemoglobin (g/dl)^**a**^	11.3 (10.6–12.2)	10.5 (9.6–11.1)	** < 0.001**
** Anemia n/N (%)**	52/169 (30.8%)	38/55 (69.1%)	** < 0.001**
Fibrinogen (mg/dl)^**b**^	412.9 (156.1)	514 (163.1)	**0.005**
** High fibrinogen level n/N (%)**	100/163 (61.3%)	35/42 (83.3%)	**0.01**
ESR (mm/h)^**a**^	32 (14–55.5)	66.5 (48.5–89.7)	** < 0.001**
** High ESR level n/N (%) (> 10 mm/h)**	137/164 (83.5%)	53/54 (98.1%)	**0.004**

*MIS-C* multisystem inflammatory syndrome in children, *KD* Kawasaki disease, *WBC* white blood cell, *ESR* erythrocyte sedimentation rate, *CI* confidence interval, Lymphopenia: < 2000 cells/µl in patients < 6y and < 1500 cells/µl in patients ≥ 6y; Thrombocytopenia: < 150 cells

^*^1000/µl; Anemia: based on age-specific level of hemoglobin [11]; Fibrinogen normal range: 150–350 mg/dl; a: expressed as median (Q1-Q3); b: defined as mean (SD)

**Fig. 2:**
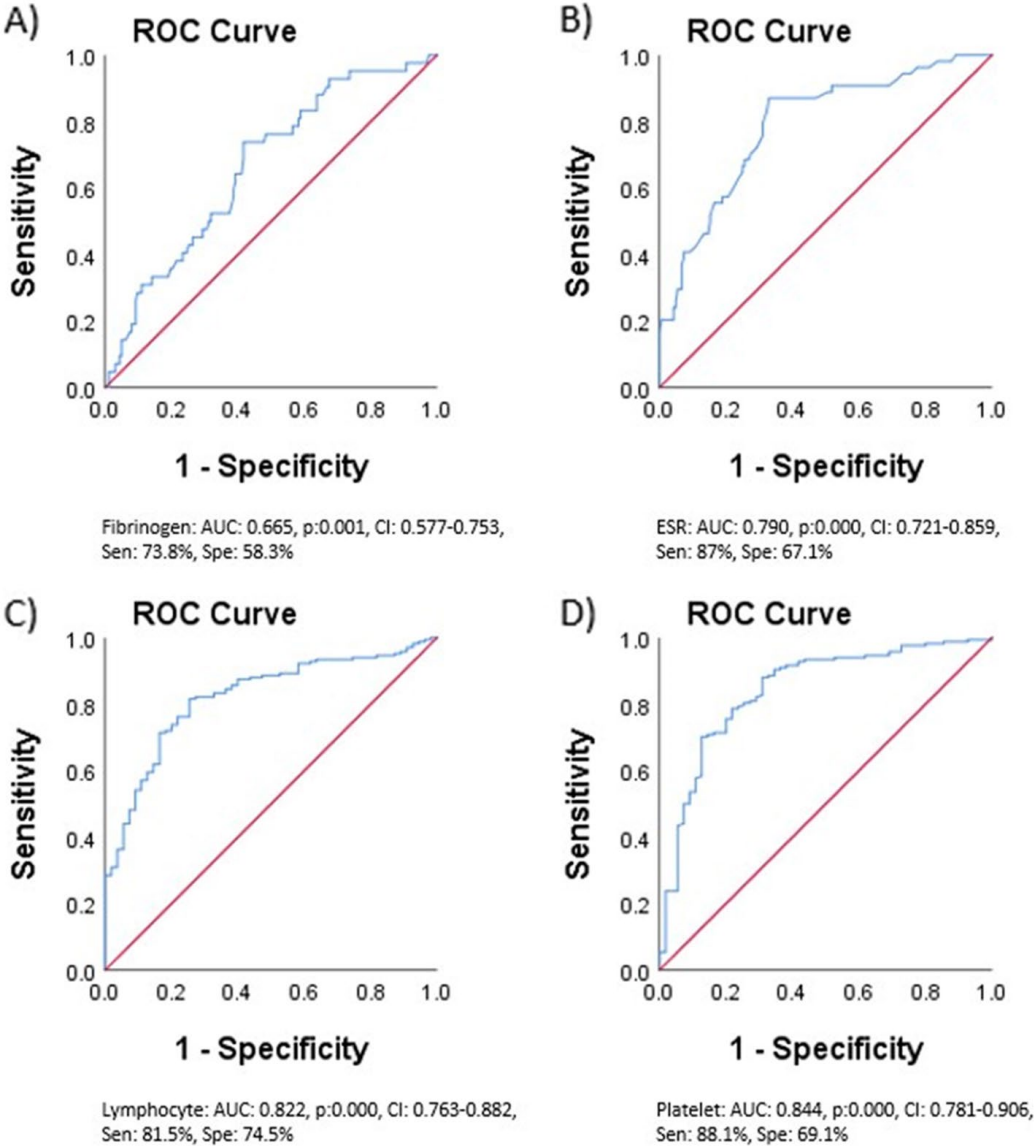
ROC curves of fibrinogen and ESR levels, and numbers of lymphocytes and platelets in predicting patients' diagnoses. Sen: sensitivity, Spe: specificity, AUC: area under the curve, *P*: *P*-value, CI: confidence interval

Using a receiver operating characteristic (ROC) analysis, the optimal cut-off values for fibrinogen and ESR levels, and the number of lymphocytes and platelets associated with the KD and MIS-C diagnoses were calculated. According to the results, a fibrinogen level of more than 433.5 mg/dl and an ESR level higher than 41.5 mm/h were diagnostic for KD. Correspondingly, lymphocyte counts lower than 3532 cells/µl and platelet counts under 372 cells/µl are associated with MIS-C diagnosis. The associated curves are illustrated in Fig. 2.

### Cardiac investigation of studied groups

The significant echocardiographic findings of patients are depicted in Table 2.

**Table 2:** Echocardiographic findings in patient groups

	MIS-C** (*N* = 176)**	KD** (*N* = 56)**	***P*-value**
**Echocardiographic findings**			
EF %^*^	62.4% (6.4)	64.4% (1.7)	**0.024**
** Low EF (< 60%), n (%)**	27 (15.3%)	1 (1.8%)	**0.004**
** PE, n (%)**	16 (9.1%)	0	**0.014**
Aneurysm, n** (%)**	2 (1.1%)	7 (12.5%)	**0.001**
Ectasia, n** (%)**	36 (20.5%)	22 (39.3%)	**0.007**

*EF* ejection fraction, *PE* pericardial effusion, *CI* confidence interval

^*^expressed as mean (SD)

Both aneurysms and ectasia were significantly less prevalent among MIS-C patients. The odds ratio (OR) for presenting an aneurysm was 12.42, which means KD patients were 12.42 times more likely to exhibit an aneurysm in their echocardiogram. Moreover, the chances of detecting ectasia were 2.51 times higher among KD patients than in the MIS-C group (OR = 2.51).

### Abdominal ultrasound findings

One hundred fifty-five patients underwent abdominal US, the findings of which are illustrated in Table 3.

**Table 3:** Abdominal US findings among patient groups

	MIS-C** (*N* = 110)**	KD** (*N* = 45)**	***P*-value**
**US** **findings**			
** Ascites**, n** (%)**	27 (24%)	2 (4%)	**0.005**
Lymphadenopathy, n** (%)**	11 (10%)	1 (2%)	0.071
Both^*^, n** (%)**	4 (3%)	0	0.999

^*^refers to exhibiting both ascites and lymphadenopathy in the abdominal US

### Post-treatment echocardiographic findings in patient groups

All patients hospitalized for more than seven days (13 in the KD group and 67 in the MIS-C group) underwent a second echocardiography seven days after receiving their first treatment based on AAP guidelines on MIS-C patients' follow-up [12]. Table 4 illustrates the significant results. Comparing the echocardiograms between the two groups, the mean EF values and the percentage of cases with EF values lower than 60% did not show any significant differences between groups.

**Table 4:** Post-treatment echocardiographic findings in studied patients

	**MIS-C (*N* = 67)**	**KD (*N* = 13)**	***P*-value**
**Echocardiographic findings**			
Aneurysm^**a**^	2/67 (3%)	3/11 (27.3%)	**0.018**
Ectasia^**a**^	11/67 (16.4%)	7/12 (58.3%)	**0.004**

^*a*^defined as cases of the event/all cases in the group

The comparisons between the two echocardiograms within each group are shown in Table 5.

**Table 5:** Comparison of pre- and post-treatment echocardiographic findings in patient groups

	**MIS-C (*N* = 67)**			**KD (*N* = 13)**		
	**Pre-treatment**	**Post-treatment**	***P*-value**	**Pre-treatment**	**Post-treatment**	***P*-value**
**Echocardiographic findings**						
EF^**a**^**(%)**	60.4% (8.4)	63.7% (3.5)	** < 0.001**	63.3% (3.0)	64.7% (0.8)	0.676
Low EF (< 60%)^**b**^	17/67	4/67	** < 0.001**	1/13	0/13	-
PE^**b**^	12/67	5/67	**0.039**	0	0	-
Aneurysm^**b**^	2/67	2/67	1	6/11	3/11	0.250
Ectasia^**b**^	26/67	11/67	**0.001**	10/12	7/12	0.375

*CI* confidence interval

^a^expressed as mean (SD)

^b^defined as cases of the event/all cases in the group

All 59 patients who exhibited CAAs in their first echocardiogram were followed for one year. CAAs remained in only six cases (~ 10%), all of whom had mild aneurysm and/or ectasia with normal EF values (mean = 64.3, SD = 1.6) in their one-year follow-up echocardiogram. One of these cases was from the MIS-C group, and the others had the diagnosis of KD.

**Fig. 3:**
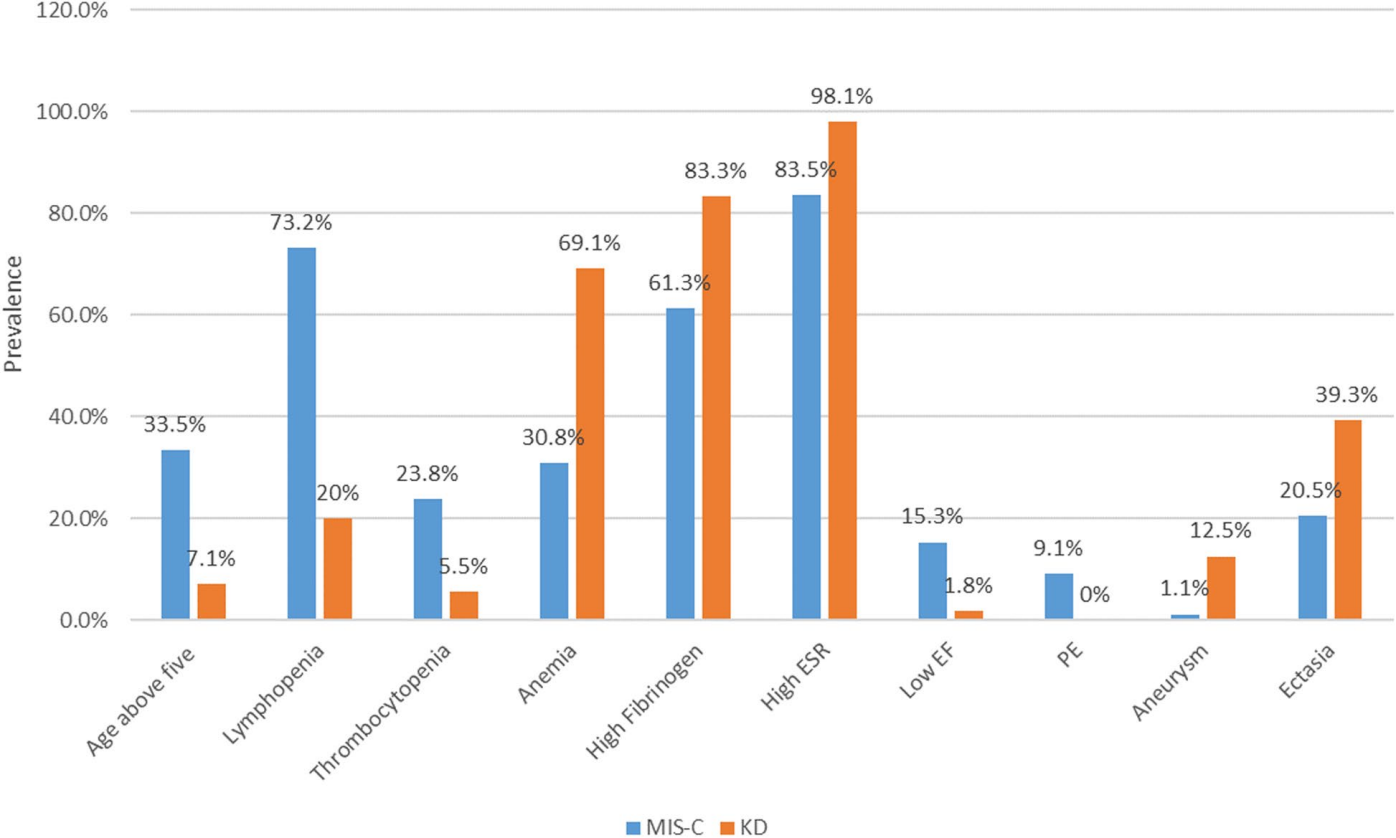
Visualization of the comparison between the two patient groups regarding their most significant differences. EF: ejection fraction, PE: pericardial effusion

Some of the most significant differences between the two studied groups are illustrated in Fig. 3.

## Discussion

To our knowledge, this is the first study to compare the echocardiographic, laboratory, and abdominal ultrasound findings, treatment outcomes, and one-year follow-up results of MIS-C and KD patients in Iran, as well as among Middle Eastern countries. Studies suggest an association between MIS-C and racial, environmental, or socioeconomic factors, and the lack of studies on MIS-C in Asian and African countries warrants investigation of this issue in countries like Iran [13]. Additionally, our relatively high number of cases allows us to draw reliable conclusions. We aim to provide clinicians with information on the distinctive features of these two entities and report patients' short-term outcomes and one-year follow-up results.

It is crucial to note that although MIS-C is believed to be associated with the epidemiological trend of SARS-CoV-2 infection, a direct link between them is yet to be confirmed [14]. More than 40% of patients with MIS-C have no evidence of SARS-CoV-2 infection (PCR and/or IgG negative) [13]. Therefore, long-term epidemiological studies are needed to confirm the suspected appearance of MIS-C along with COVID-19 outbreaks.

MIS-C is a heterogeneous entity, with significant overlap in features with other hyper-inflammatory syndromes in children (e.g., toxic shock syndrome, macrophage activation syndrome, and KD). This overlap may explain the low sensitivity and specificity of different inflammation markers to distinguish these diseases as they share similar dysregulations in inflammatory pathways [13].

Our results suggest that MIS-C affects children of older age groups than KD. In our patient groups, the median age of MIS-C patients is nearly three times as high as the median age of the KD group. This result is consistent with other studies [15–17]. In contrast to the male predominance in KD [18], there are still controversies about gender predominance among MIS-C patients [18–20]. The analysis of our cases indicates no gender dominance either in MIS-C or KD.

Turning to laboratory work-up, MIS-C is shown to be associated with lower WBC counts and lymphopenia, whereas leukocytosis is known to be a distinct feature in KD [8]. This difference is stated in all previous studies and is considered one of the main domains through which these two entities can be distinguished. The pathophysiology underlying the lymphopenia in MIS-C is deemed to be the same as that in COVID-19 infection, which is immune exhaustion. The lymphopenia caused by COVID persists long after the illness and is observed in individuals displaying MIS-C, which is assumed to be a complication of COVID-19 [18, 21–23]. Platelet count is another distinctive factor. While thrombocytosis is a constant finding among KD patients [8], thrombocytopenia is repeatedly seen in MIS-C. Yeo et al. explain that COVID infection results in bone marrow suppression, exhibiting thrombocytopenia. Conversely, immune complexes in untreated KD would initiate platelet recruitment and cause thrombocytosis [19, 22, 24]. Anemia is reported to be more prevalent among KD patients [16, 21]. Correspondingly, in our study, the prevalence of anemia was more than twice as high in KD as in MIS-C. Active inflammation and the iron deficiency caused by hepcidin can explain our results [25].

Regarding inflammatory markers, the ESR level is reported to rise significantly among MIS-C and KD patients. Despite a significant ESR rise in both groups, high ESR levels were more prevalent in KD than in the MIS-C group [7, 19, 21]. Another inflammatory marker that is widely discussed in this subject is CRP. Although CRP level is reported to increase in both diseases, the difference in CRP levels within these two groups being of statistical or clinical significance is still controversial [15, 16, 19]. Our results suggest a considerable rise in CRP levels in both groups of patients without any distinctive differences between them.

Like CRP, the variability of fibrinogen levels among the KD and MIS-C groups is the subject of a discrepancy between various studies [7, 19, 22]. Our investigation suggests a noticeable increase in fibrinogen levels in both groups as an acute phase reactant, with KD patients exhibiting relatively higher levels. Nevertheless, more studies are required to determine whether these two entities can be distinguished using fibrinogen levels. In contrast to previous studies, which suggest higher levels of ferritin in MIS-C compared to KD [7, 19, 26], our data do not support this discrepancy. In our studied patients, the ferritin level is slightly higher in KD; however, it is not statistically proven to be a considerable difference between the two patient groups.

Other laboratory indices (LDH, CPK, CK-MB, and troponin I) being tested in this study show no significant discrepancies among KD and MIS-C patients. LDH level is insignificantly higher in MIS-C. Although both groups display increased LDH levels, none of the studies have proposed it as a distinguishing factor between MIS-C and KD [18, 27, 28]. This rise can implicate organ damage, a case-defining criterion for both entities. The figures for CPK and CK-MB levels did not differ among patient groups.

Regarding the cardiovascular aspects of the studied patients, previous investigations have reported higher rates of LV dysfunction, myocardial involvement, pericarditis, valvular regurgitation, and pericardial effusion among MIS-C patients compared to the KD group. On the other hand, CAAs are shown to be more pronounced in KD [7, 16, 19]. Our results suggest a higher prevalence of pericardial effusion among MIS-C patients, while CAAs are less likely in them. The proposed difference can be utilized to distinguish the two entities. There was a slight but significant decrease in the mean ejection fraction in the MIS-C group compared to KD. Accordingly, 15.3% of our MIS-C group revealed EF values less than 60%, which was only 1.8% in the KD group. This result supports the LV dysfunction in MIS-C proposed by previous studies. There were no cases of pericarditis, and only two cases of myocarditis and valvular insufficiency among our patients, which could not lead to any conclusion.

Among the clinical symptoms reported in patients, abdominal pain is more likely to be reported by those affected by MIS-C. Furthermore, some patients have undergone laparotomy with the impression of acute abdomen, which was later found to be incorrect [15, 16, 19]. However, consistent with previous studies, our research reveals a higher prevalence of mild ascites in MIS-C, which can explain the higher incidence of gastrointestinal symptoms in MIS-C compared to KD [29–32].

Regarding short-term outcomes in our patients, there was a significant improvement in the mean EF after treatment in the MIS-C group. Furthermore, the percentage of PE and ectasia in this group was considerably lower one week after the initiation of therapy. The number of patients exhibiting aneurysms decreased from 8 to 5, and no new cases of aneurysms were observed in their second echocardiogram. However, this reduction is not statistically significant, and further follow-up is required. Our results suggest that the higher prevalence of coronary artery aneurysms and ectasia among KD patients compared to the MIS-C group persists even after seven days of treatment initiation, indicating long-term coronary complications in KD. The persistence of CAAs in 6 cases one year after the first admission, who were mostly KD patients exhibiting both aneurysm and ectasia, warrants further follow-up for these patients. Although the higher prevalence of persisting CAAs among KD patients after a year can be attributed to the higher initial incidence of CAAs in KD compared to MIS-C, there might be other underlying mechanisms that accelerate CAA recovery in MIS-C patients. This issue requires further investigation.

## Conclusions

In conclusion, this study contributes to our understanding of MIS-C and KD by analyzing a substantial number of patients and comparing their laboratory and clinical features, as well as short-term outcomes and one-year follow-ups. The results highlight important differences between MIS-C and KD, with MIS-C patients exhibiting characteristics such as lymphopenia, thrombocytopenia, lower ESR and fibrinogen levels, along with specific findings like ascites, lower EF, PE, and a lower incidence of CAAs. These distinguishing factors can aid in accurate diagnosis and differentiate MIS-C from KD, despite their overlapping symptoms. The findings from this study contribute to the existing body of knowledge and provide clinicians with valuable insights for improved management and care of pediatric patients presenting with these conditions.

## Limitations

Our study has several limitations. Laboratory findings such as WBC and differentials, hemoglobin, and platelet counts are known to be influenced by the age of the children. Additionally, CRP and inflammation-associated indices can be affected by the stage of the disease. Therefore, further studies with a larger study population are needed to adjust for age and disease stage.

Within one year of our patient recruitment, various strains of SARS-CoV-2, including Beta, Delta, and Omicron, have affected our country. Given the different manifestations of infection with these variants, it is inevitable that not all our cases would present with identical laboratory and echocardiographic findings. Therefore, reporting the results as mean or median values may be misleading. This could explain the lack of significant differences observed between the two groups of patients after comparing some indices. Additionally, some cases meet the diagnostic criteria for both KD and MIS-C. Therefore, some patients can be diagnosed with both KD and MIS-C simultaneously.

## Data Availability

All data generated or analyzed during this study are available from the corresponding author upon reasonable request.

## Abbreviations

CDC: Centers for Disease Control and Prevention
COVID-19: Coronavirus disease 2019
CRP: C-reactive protein
EF: Ejection fraction
ESR: Erythrocyte sedimentation rate
KD: Kawasaki disease
MIS-C: Multisystem inflammatory syndrome in children
PE: Pericardial effusion
SARS-CoV-2: Severe acute respiratory syndrome coronavirus type2
US: Ultrasound
WHO: World health organization
